# Complementary oligonucleotides regulate induced fit ligand binding in duplexed aptamers†
†Electronic supplementary information (ESI) available. See DOI: 10.1039/c6sc03993f



**DOI:** 10.1039/c6sc03993f

**Published:** 2016-12-08

**Authors:** Jeffrey D. Munzar, Andy Ng, Mario Corrado, David Juncker

**Affiliations:** a McGill University and Genome Quebec Innovation Centre , 740 Dr. Penfield Avenue , Montreal , Quebec H3A 0G1 , Canada . Email: david.juncker@mcgill.ca; b Department of Biomedical Engineering , McGill University , 3775 Rue University , Montreal , Quebec H3A 2B4 , Canada; c Department of Neurology and Neurosurgery , McGill University , 3801 Rue University , Montreal , Quebec H3A 2B4 , Canada

## Abstract

Duplexed aptamers (DAs) are engineered by hybridizing an aptamer-complementary element (ACE, *e.g.* a DNA oligonucleotide) to an aptamer; to date, ACEs have been presumed to sequester the aptamer into a non-binding duplex state, in line with a conformational selection-based model of ligand binding. Here, we uncover that DAs can actively bind a ligand from the duplex state through an ACE-regulated induced fit mechanism. Using a widely-studied ATP DNA aptamer and a solution-based equilibrium assay, DAs were found to exhibit affinities up to 1 000 000-fold higher than predicted by conformational selection alone, with different ACEs regulating the level of induced fit binding, as well as the cooperative allostery of the DA (Hill slope of 1.8 to 0.7). To validate these unexpected findings, we developed a non-equilibrium surface-based assay that only signals induced fit binding, and corroborated the results from the solution-based assay. Our findings indicate that ACEs regulate ATP DA ligand binding dynamics, opening new avenues for the study and design of ligand-responsive nucleic acids.

## Abstract 2

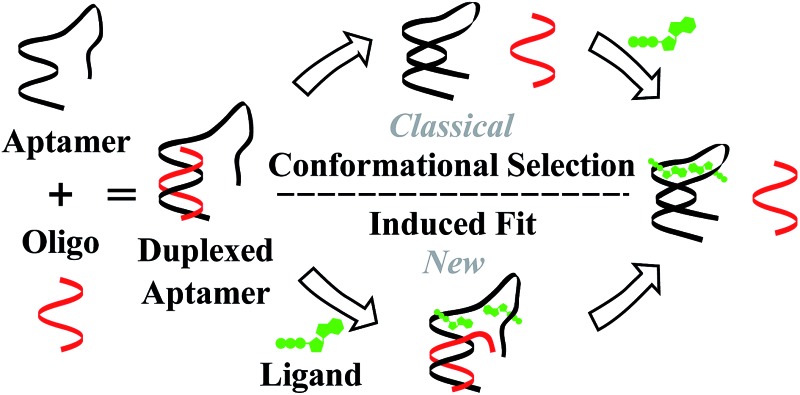
Hybridizing a complementary oligonucleotide to an ATP aptamer is shown to functionally regulate a newly revealed induced fit ligand-binding pathway.

## Introduction

**Fig. 1:**
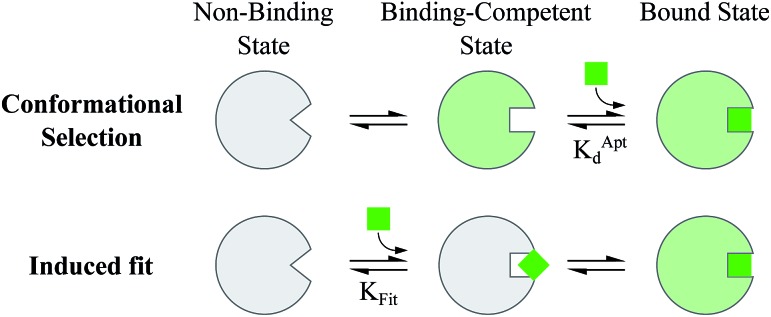
Ligand binding pathways in nature. In conformational selection, the binder shifts between distinct conformational states, and the ligand only stabilizes a pre-existing binding competent state. Induced fit describes a binding pathway in which the ligand catalytically reorganizes the binder into a favorable conformation, leading to the ligand bound state.

Proteins and functional nucleic acids often couple specific ligand-binding events with distinct structural changes central to biochemical regulation.^1,2^ Likewise, synthetic nucleic acid aptamers undergo distinct structural changes upon ligand binding, which can be leveraged for biosensing and applications in synthetic biology.^3–9^ The binding-related structural changes that occur in these ligand-binder systems can be described as proceeding *via* either conformational selection (MWC model^10^) or induced fit (KNF model^11^) (Fig. 1). In conformational selection, a binder exists at equilibrium between non-binding and binding-competent states, and is only capable of binding the ligand in the latter. Alternatively, induced fit describes a binding pathway from the non-binding to bound state actively catalyzed by the ligand.

Interestingly, aptamers can be engineered with enhanced switching activity by hybridizing an aptamer-complementary element (ACE, such as a short DNA oligo) to a desired aptamer sequence, forming a duplexed aptamer (DA) that acts as a synthetic switch.^12–15^ The ease of engineering DAs from known aptamer sequences has led DAs to find numerous applications based on *e.g.* FRET,^12^ electrochemistry,^16–22^ colorimetry,^23^ SPR,^24^ fluorescence,^25,26^ and signaling cascades.^27^

However, to date, ligand binding in DAs has only been modeled based on conformational selection, in which the ACE acts as an inhibitor, sequestering the aptamer into a non-ligand-binding, passive duplex state. In this model, the observed affinity of a DA (*K*Obsd) is a function of (i) the intrinsic affinity of the native aptamer (*K*Aptd) and (ii) the hybridization free energy of the ACE-aptamer duplex (*K*_Hyb_).^28–36^ In qualitative agreement with such a model (for details, see Fig. S1†), Porchetta *et al.* used a native cocaine DNA aptamer and varying length ACEs (10–15 bases) to engineer and tune the relative affinity of cocaine DAs over three orders of magnitude.^32^ To our knowledge, although concepts of 3-body side reactions and misfolded sensor states have been used to model DAs,^28^ induced fit ligand binding in DAs, in which a DA might actively sense and catalytically bind a ligand directly from the duplex state, has not been studied. In this regard, we note that small modifications to the length and location of ACEs have been documented to impact DA biosensors in ways that cannot be accounted for by differences in ACE-aptamer hybridization free energies.^37–39^

**Fig. 2:**
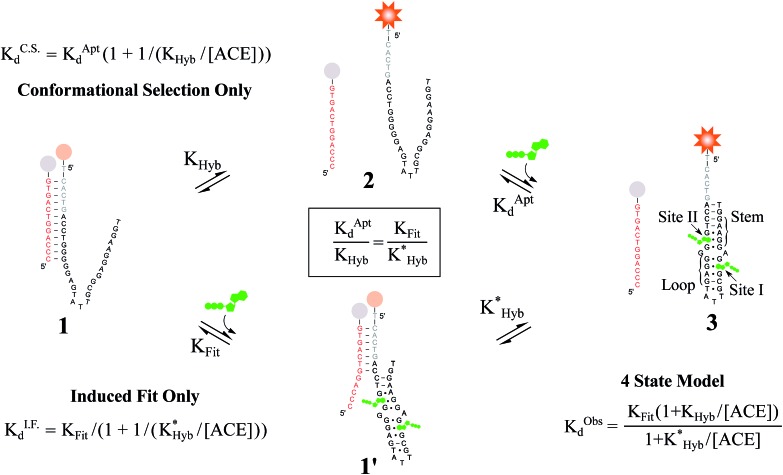
Overview of ligand binding in duplexed aptamers. The 5′Q_-5C:12_ DA can be described by an equilibrium of four representative states and two binding pathways. The consensus aptamer sequence (black) and 5′Q_-5C:12_ ACE (red) are shown as an example, with canonical (dashes) and non-canonical (dots) base pairs included. The ATP binding sites and stem and loop regions of the native aptamer are labeled for state 3. The binding affinity of a DA is a function of the conformational selection pathway, governed by the duplex hybridization free energy (*K*_Hyb_) and the apparent aptamer affinity (*K*Aptd), as well as the proposed ATP-dependent induced fit binding pathway, which is governed by the induced fit binding affinity of the DA (*K*_Fit_) and the hybridization free energy of the ligand-disrupted duplex 
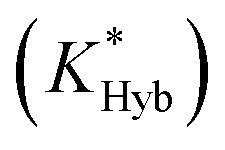
. Derivations of the analytical models for these two pathways (*K*C.S.d, *K*I.F.d) and the four-state model are provided in the ESI.†

Here, we evaluate the possibility of induced fit ligand binding in DAs. We focused on DAs engineered from the Huizenga and Szostak ATP DNA aptamer introduced in 1995,^40,41^ as this aptamer is the most widely studied, is well characterized, and was the first to be implemented as a DA.^12^ The native aptamer binds ATP (6 μM *K*Aptd) first through folding of the stem and loop regions, followed by the cooperative binding of two ATP molecules within the binding pocket (sites I and II, Fig. 2).^40–43^ Using DAs engineered from the ATP aptamer, we first performed equilibrium solution-based assays and uncovered (i) the existence of induced fit ligand binding in ATP DAs, and (ii) that ACEs allosterically regulate induced fit ligand binding, thereby modulating the affinity of ATP DAs in an unexpected manner. To confirm these findings, we performed a second set of experiments using a non-equilibrium surface-based assay that we developed.

## Results and discussion

**Fig. 3:**
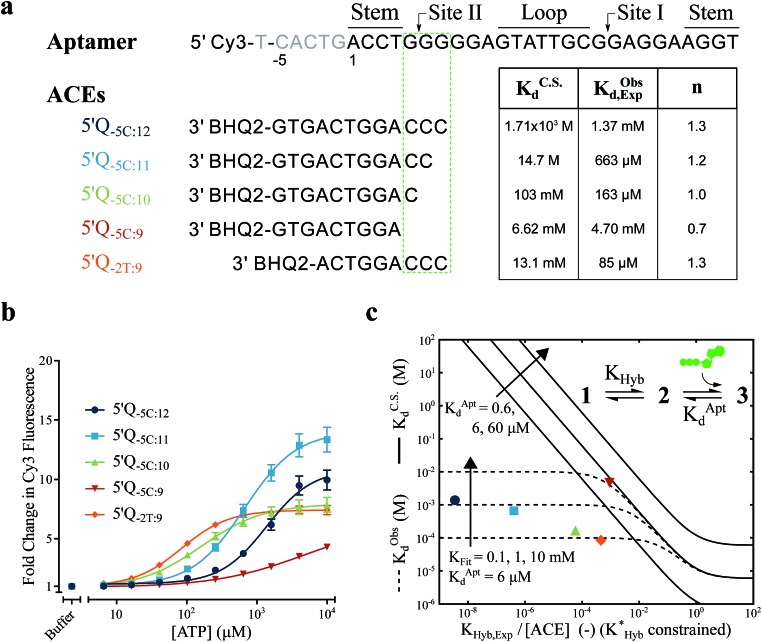
Results and analytical modeling of a solution-based FRET assay for 5 ATP DAs. (a) Aptamer and ACE sequences tested, showing overlap of studied ACEs with binding site II, together with a table of DA binding affinities predicted by conformational selection only (*K*C.S.d), as well as experimentally measured DA binding affinities (*K*Obsd,Exp) and cooperativities (*n*) for all 5 DAs. (b) Solution-based FRET assay results. Lines are 4 parameter fits used to calculate *K*Obsd,Exp and *n*; error bars represent ±1 S.D. (*N*_replicates_ = 3). (c) Graph showing experimental results and analytical models of DA observed affinities as a function of experimentally measured duplex hybridization free energies (*K*_Hyb,Exp_/[ACE]). Solid lines represent a conformational selection-only model (*K*C.S.d isolines, for varying *K*Aptd); dashed lines represent a model accounting for both conformational selection and induced fit pathways (*K*Obsd isolines, for varying *K*_Fit_); experimental data as in (b and c). Whereas 5′Q_-5C:9_ (red triangle) falls in good agreement with a conformational selection analytical model, the four other DAs exhibited much higher binding affinities that are only accounted for by a model including ACE-regulated induced fit.

For the solution-based assay, ATP DA constructs were designed starting with a 32-mer Cy3-labeled variant of the ATP aptamer, together with a previously reported 12-mer ACE, 3′-labeled with a BHQ-2 quencher.^12^ This ACE hybridizes to 12 bases at the 5′ end of the aptamer, beginning five bases outside the consensus aptamer sequence at the -5C nucleotide, and is termed 5′Q_-5C:12_. Representative states in the candidate binding pathways for the 5′Q_-5C:12_ DA are shown in Fig. 2. To assess any impact of ACE length and location on DAs, we engineered DAs using successively 5′-truncated 5′Q_-5C:12_ ACEs, as well as a three-base 3′-truncated ACE (Fig. 3a). After confirming duplex formation for all five ACEs (Fig. S2†), ATP DAs were tested using an equilibrium solution-based FRET assay^29,32^ (see ESI methods†), with ACE:aptamer (Q:F) ratios of 1:1 (Fig. 3b) and 3:1 (Fig. S3†).

For a 1:1 Q:F ratio, the experimentally observed affinities of the 5 DAs are poorly predicted using a conformational selection-based analytical model of DAs (Fig. 3a and c). Based on experimentally measured hybridization DA free energies (obtained using FRET melting,^44^ see ESI methods, Table S1 and Fig. S5†), only the 5′Q_-5C:9_ data is consistent with a conformational selection model (Fig. 3c; *K*C.S.d, *K*Aptd = 6 μM solid isoline), while the four other DAs tested are not. The analytical model underestimates 5′Q_-5C:12_ affinity by more than 1 000 000-fold, and predicts a 15-fold increase in apparent affinity for 5′Q_-5C:9_ over 5′Q_-5C:10_, whereas a 28-fold decrease in affinity was observed (Fig. 3a and c).

An analytical model incorporating an induced fit binding pathway alongside conformational selection (and in which each DA has an ACE-dependent induced fit binding affinity (*K*_Fit_)) can account for the unexpectedly high affinity of these DAs (Fig. 3c; see ESI derivation and Fig. S4† for additional modeling). This model suggests that induced fit binding is limited in the 5′Q_-5C:9_ DA (*K*_Fit_ > 10 mM, Fig. 3c), whereas the four other DAs exhibit *K*Obsd,Exp in agreement with *K*_Fit_ values of 100–1000 μM.

Additionally, the ACEs tested gave rise to DAs with differing ATP binding cooperativities, as determined based on the Hill slope, *n*, suggesting that ACEs also modulate DA allostery. Here, reduced interaction of ACEs with site II promoted a shift from cooperative to anti-cooperative binding for Q:F ratios of 1:1 (*n* = 1.3 to 0.7) and 3:1 (*n* = 1.8 to 0.9) (Fig. 3a, b and S3†). Interestingly, positive cooperativity of the DA was restored using the site II-hybridizing 5′Q_-2T:9_ ACE (*n* = 1.3 and 1.7 for 1:1 and 3:1 Q:F ratios), suggesting that ACEs hybridized to site II yield DAs capable of cooperative ligand binding, as present in the native aptamer (*n* = 2.0 (ref. 42)).

**Fig. 4:**
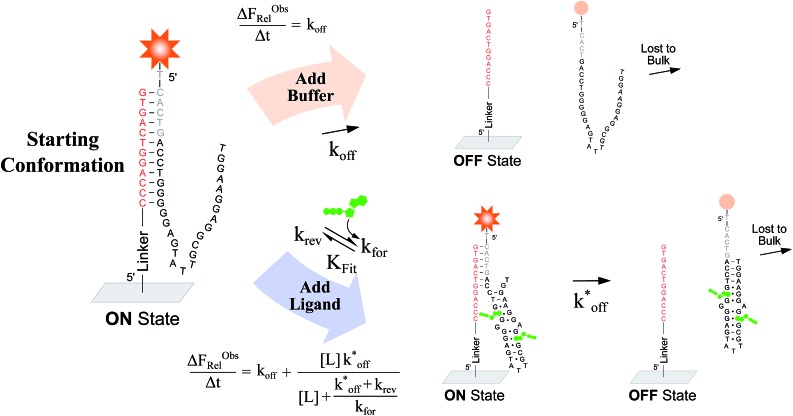
Overview of a surface-based fluorescence assay used to measure the induced fit ligand binding affinity of a DA. A fluorescently labeled aptamer is hybridized to covalently immobilized ACEs in replicate wells, and incubated for a set time (Δ*t*) with buffer, or with buffer and ligand. The baseline dissociation rate (*k*_off_) of DAs is quantified by measuring the relative loss of fluorescent aptamer (Δ*F*ObsRel/Δ*t*) following incubation with buffer only. In parallel, the increase in Δ*F*ObsRel/Δ*t* arising from incubations with increasing ligand concentrations is measured, from which the induced fit binding affinities of DAs (*K*_Fit,Exp_) can be calculated.

To verify the unexpected findings obtained from the solution-based assay, we developed a surface-based assay that signals only when a DA binds a ligand *via* induced fit, and in which conformational selection plays no role (Fig. 4). In contrast with the solution-based FRET assay, the surface-based fluorescence assay does not operate at equilibrium. Here, fluorophore-conjugated aptamers are first hybridized to ACEs covalently coupled on a slide surface, followed by washing off of non-hybridized aptamers, yielding surface-immobilized DAs (Fig. 4). After incubation with buffer (or buffer and ligand) for a specified time (Δ*t*), DA dehybridization is measured as a loss of surface fluorescence (Δ*F*ObsRel); owing to the low concentration of released aptamers, DA dissociation is effectively non-reversible. By assaying varying ligand concentrations, the surface-based fluorescence assay can be used to derive the induced fit affinity (*K*_Fit_) of a DA.

**Fig. 5:**
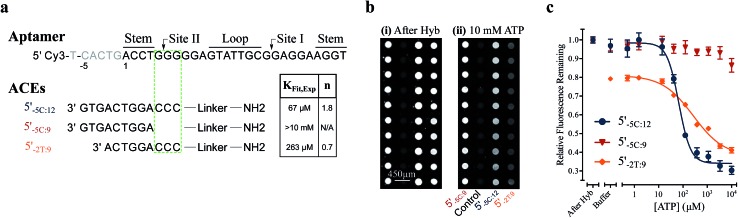
Results of a surface-based DA fluorescence assay. (a) Sequences of the aptamer and ACEs tested, and experimentally derived *K*_Fit,Exp_ and cooperativities (*n*). (b) Fluorescence images of the surface-based assay for three ACEs after (i) hybridization and (ii) 1 h incubation with 10 mM ATP. (c) Changes in relative fluorescence with ATP titration for three ATP DAs. Lines represent 4 parameter fits used to calculate *K*_Fit,Exp_ and *n*; error bars represent ±1 S.E. (*N*_experiments_ = 4). The 5′Q_-5C:9_ DA is found to be insensitive to ATP, whereas the ATP-dependent fluorescence responses for 5′Q_-5C:12_ and 5′Q_-2T:9_ DAs reveal induced fit binding.

We used this surface-based assay to study ATP DAs constructed from three ACEs, corresponding to three ACEs used in the solution-based FRET assay (but missing quenchers). These ACEs varied in length and degree of site II hybridization to the ATP aptamer, termed 5′_-5C:12_, 5′_-5C:9_, and 5′_-2T:9_ (Fig. 5a). A 3-plex microarray was constructed with the 3 ACEs, incubated with Cy3-labeled aptamer, briefly washed, and imaged with a fluorescence scanner (ESI methods†) to establish a baseline. Next, sub-arrays on the microarray were incubated for 1 h each with ATP in buffer, or with buffer only, followed by a second fluorescence scan (Fig. 5b). Given the high concentrations of ATP assayed, the low dissociation rate of modified duplexes 
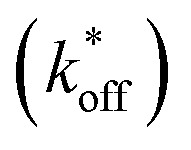
 expected for the ACEs tested here (Table S1†), and assuming a steady state of intermediate duplexes on the surface, this assay can be modeled by Briggs-Haldane kinetics. By also assuming 
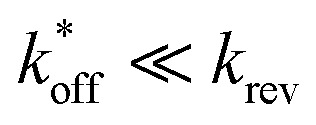
 (Fig. 4), the experimental induced fit binding affinity of a DA (*K*_Fit,Exp_) is equal to the Michaelis constant derived from Δ*F*ObsRel with ATP titration (Fig. 5c).

The surface-based fluorescence assay yielded a high induced fit binding affinity for the site-II hybridizing 5′_-5C:12_ ATP DA (*K*_Fit,Exp_ of 67 μM) (Fig. 5a and c). However, the 5′_-5C:9_ DA, which shares a footprint with 5′_-5C:12_ but with site II left unhybridized, displayed no induced fit binding (*K*_Fit,Exp_ > 10 mM, Fig. 5a and c), consistent with the solution-based FRET assay findings (Fig. 3a, c and S4†). Thus, despite having a much lower hybridization free energy than the 12-mer ACE, the 9-mer 5′_-5C:9_ ACE does not promote induced fit. Meanwhile, DAs engineered with the site-II hybridizing 5′_-2T:9_ ACE displayed a *K*_Fit,Exp_ of 263 μM (Fig. 5a and c). This value is in good agreement with an analytical model of the solution-based assay including both conformational selection and induced fit binding pathways (Fig. 3c and S4†). As a negative control ligand, we also assayed microarrays with GTP, and no induced fit binding was observed (Fig. S6†).

As observed in the solution-based FRET assay for site-II hybridizing ACEs, the 5′_-5C:12_ ACE also formed a DA with positive cooperativity for ATP (*n* = 1.8) (Fig. 5a and c). Interestingly, the 5′_-2T:9_ DA displayed no cooperativity (*n* = 0.7); given the unstable duplex expected for an ATP-disrupted 5′_-2T:9_ DA (Table S1†), this result may indicate that a single ATP is sufficient to displace the 5′_-2T:9_ ACE in the surface-based assay. Overall, the surface-based assay results corroborate the solution-based assay findings, supporting an ACE-dependent induced fit binding mechanism in ATP DAs. These findings also suggest that the ACE-based allosteric regulation of ATP DAs is relatively independent of biosensor design.

## Conclusions

Taken together, our results indicate that induced fit ligand binding can be a dominating pathway in DAs, and that single nucleotide changes to ACEs can significantly modify ATP DA induced fit binding dynamics. These findings point towards ACEs as underappreciated functional regulators of the binding affinity, binding cooperativity, and allostery of DAs. This work opens new avenues for tuning aptamer-based systems, with particular relevance to the use of aptamers in biosensing and synthetic biology. However, it is not yet clear if induced-fit ligand binding in DAs is rare, with the family of ATP DAs studied here being an exception to the rule, or if induced fit is a general binding pathway common to duplexed ligand-responsive nucleic acids. In this regard, the non-equilibrium surface-based fluorescence assay introduced here could be expanded to investigate additional ACEs, to test other ACE-aptamer combinations, to investigate the effect of changes to structural regions of an aptamer on DAs (such as modifying the ligand binding pocket or tertiary structure-stabilizing bases), or to investigate other functionalities played by ACEs in DAs, such as perturbing ligand specificity.^45,46^

Finally, this work also highlights the ACE-specific regulation of DA ligand binding as a novel model of the thermodynamic and structural determinants that govern transitions between ligand binding pathways. In this sense, we note that DAs may offer researchers a uniquely tractable and configurable nucleic acid-based alternative to existing protein-based models of allosteric regulation^47–49^ and collective motion in biopolymers.^50–52^
