# Supplementary material for: Complementary oligonucleotides regulate induced fit ligand binding in duplexed aptamers
Source: Chem Sci. 2016 Dec 8;8(3):2251–6. doi: 10.1039/c6sc03993f (PMC5408566; doi:10.1039/c6sc03993f)
Supplement: Supplementary file 1 [file SC-008-C6SC03993F-s001.pdf]

Electronic Supplementary Material (ESI) for Chemical Science.
This journal is © The Royal Society of Chemistry 2016

Supporting Information

Complementary Oligonucleotides Regulate Induced Fit Ligand Binding in Duplexed Aptamers

Jeffrey D. Munzar[ab], Andy Ng[ab], Mario Corrado[ab], and David Juncker[*abc]

[a] McGill University and Genome Quebec Innovation Centre, 740 Dr. Penfield Avenue, Montreal, Quebec H3A 0G1, Canada

[b] Department of Biomedical Engineering, McGill University, 3775 Rue University, Montreal, Quebec H3A 2B4, Canada

[c] Department of Neurology and Neurosurgery, McGill University, 3801 Rue University, Montreal, Quebec H3A 2B4, Canada

[*] Corresponding author: david.juncker@mcgill.ca

Table of Contents

Reagent Information

Detailed Experimental Methods

Analytical Model Derivations

Tab. S1 Measured and simulated thermodynamic properties of ACEs tested in this study.

Tab. S2 Complete sequences of DNA oligonucleotides used in this study.

Fig. S1 Graphical representation and modeling of observed binding affinities for cocaine DAs.

Fig. S2 Additional experimental results of solution-based FRET assay.

Fig. S3 Experimental results of a solution-based FRET assay using a 3:1 Q:F ratio.

Fig. S4 Additional analytical models of ATP DA observed binding affinities, as a function of simulated K_{Hyb} and K^*_{Hyb} values.

Fig. S5 FRET melting curve experimental results.

Fig. S6 Experimental (ATP) and negative control (GTP) surface-based fluorescence assay results for the $5'_{-5C:12}$ DA.

References

Reagent Information

All oligonucleotides were purchased from Integrated DNA Technologies (Coralville, Iowa, USA) and were purified by HLPC. See Table S2 for a complete list of oligos used in this study. Commercial 2D aldehyde-functionalized glass slides were purchased from PolyAn GmbH (Berlin, Germany).

Sodium chloride was purchased from Fisher Scientific (Ottawa, Ontario, Canada). Magnesium chloride, betaine, sodium citrate dihydrate, Trizma base, 10x PBS, ethanol, SDS, sodium borohydride and Tween 20 were purchased from Sigma (Oakville, Ontario, Canada). Bovine Serum Albumin (BSA) was purchased from Jackson ImmunoResearch (West Grove, Pennsylvania, USA). ATP and GTP (100 mM, pre-titrated with NaOH) were purchased from Life Technologies (Burlington, Ontario, Canada). All water used was deionized to 18 MΩ using a MilliQ system from EMD Millipore (Etobicoke, Ontario, Canada).

Detailed Experimental Methods

Solution FRET Assay. Cy3-labelled DNA aptamer and each BHQ2-quencher-labeled ACE were reconstituted to 100 μM in water, aliquoted, and stored at -20˚C. The concentration of these stocks was confirmed using a Nanodrop 2000 UV-Vis spectrometer (Nanodrop, Wilmington, Delaware, USA). For the solution FRET assays, all fluorescence measurements were carried out in a total volume of 10 μL in Assay Buffer[1] (20 mM Tris, 300 mM NaCl, 5mM $MgCl_2$, pH 8.3) containing 0.1 % Tween 20 in low-volume black 384 well non-binding surface microplates (Corning #3676, Corning, New York, USA). Tween 20 was included in all solution FRET assay experiments to minimize the loss of DNA oligos to the surface of plasticware used for handling the solutions, which can lead to erroneous standard curve measurements, Fig. S2a. We used a SpectraMax i3x multimode plate reader (Molecular Devices, Sunnyvale, California, USA) in top-read mode to quantify fluorescence, with a 550 nm wavelength fluorescence excitation source (9 nm bandwidth) and 579 nm wavelength fluorescence detector (15 nm bandwidth), maintained at 25˚C.

A linear calibration curve of Cy3 fluorescence was constructed by serially diluting Cy3-labelled aptamer in Assay Buffer from 1 μM to 200 pM, Fig. S2a. Next, duplex formation for each ACE-specific DA was validated by constructing quenching curves for each DA using a final concentration of 200 nM Cy3 aptamer and Q:F ratios of 10:1 to 0.05:1, Fig. S2b,c. To minimize pipetting errors, master stocks containing 2 μM of each quencher-labeled ACE and 200 nM of Cy3 aptamer were serially diluted using a stock of 200 nM Cy3 aptamer. After serial dilution, all solutions were heated to 72˚C for 5 min, 41˚C for 5 min, and then incubated at 25˚C for 45 min before carrying out fluorescence measurements with the plate reader.

To carry out solution FRET assays for ATP, a similar serial dilution strategy was implemented, starting with master stocks of 200 nM Cy3 aptamer combined with 200 nM (or 600 nM) of each quencher-labeled ACE. These solutions, supplemented with 10 mM ATP, were then used to perform serial dilutions, yielding final solutions containing 200 nM Cy3 aptamer, 200 nM (1:1 Q:F) or 600 nM (3:1 Q:F) ACE quencher, and 10 mM to 6.5 μM ATP. After serial dilution, all solutions were heated to 72˚C for 5 min, 41˚C for 5 min, and then incubated at 25˚C for 45 min before fluorescence readout.

FRET Melting Assay. The hybridization affinity of the solution-based DAs were measured using FRET melting (e.g. [2,3]). ACE:aptamer solutions with a 1:1 molar ratio were prepared in dilutions ranging from 4 μM to 400 nM (total strand concentrations, C_t) in Assay Buffer containing 0.1 % Tween 20 via serial dilution. Simultaneous thermal denaturation of all samples was achieved using a rotary qPCR machine (Corbett Rotor-Gene 6000, Qiagen, Valencia, California, USA) with fluorescence acquisition in the yellow channel (Excitation: 530 nm; Detection: 555 nm; Gain: 10/10). The melt curve was programmed as follows: all samples were heated to 95˚C for 5 min, then slowly cooled from 95˚C to 25˚C in steps of 0.05˚C held for 3 seconds each, then held at 25˚C for 2 min, and finally heated from 25˚C to 95˚C in steps of 0.05˚C held for 3 seconds each. When including the additional time taken by the cycler to change temperate (~3 seconds), the thermal ramp implemented here was approximately 0.5˚C/min.

To extract thermodynamic variables, melt curves for all ACE:aptamer samples were first normalized to the fluorescence of a sample containing only Cy3-labeled aptamer, which was done to account for the temperature-dependent decrease in fluorescence of Cy3. Next, T_m values were extracted using Matlab by defining the T_m as the

temperature at which a sample achieves a half-maximal normalized fluorescence intensity, with ΔH, ΔS and $\Delta G_{Hyb}^{25^\circ C}$ values extracted based a van't Hoff plot of $1/T_m$ vs. $\ln(C_t/4)$ (see Tab. S1 and Fig. S4 for details).

ACE Microarray Fabrication. ACE microarrays were printed on 2D aldehyde-functionalized glass slides using an inkjet spotter (Nanoplotter 2.0, GeSiM, Radeberg, Germany). Prior to printing, amine-functionalized ACEs were dissolved to a final concentration of 8 μM in 1 x Printing Buffer (3xSSC, 1.5 M betaine, pH 7.0) (Note: 20xSSC contained 3 M NaCl, 0.3 M Sodium Citrate, pH 7.0). Each microarray feature was made by printing a total volume of 2.0 nL onto the slide surface using a type J NanoTip (GeSiM). 10 replicate spots of each ACE were printed per slide sub-array, with up to 14 replicate subarrays printed per slide. Printing was carried out at 70 % relative humidity and 25˚C in the dark, and slides were incubated for 8 h under these conditions to ensure uniform binding of ACEs to the slide surface within each microarray feature.

After printing, ACE microarrays were washed with 0.2 % SDS for 4 min and water for 5 min. Remaining aldehyde functional groups on the slide were reduced using a 0.25 % sodium borohydride, 0.75 x PBS, 25 % ethanol buffer for 45 min, followed by a wash in 0.2 % SDS for 4 min, and a final wash in water for 5 min. After washing, slides were immediately preincubated in Hybridization Buffer (4xSSC, 1 % BSA, pH 7.0) for 1 h, washed a final time with water for 5 min, and then dried using filtered nitrogen gas. All washing, deactivation and prehybridization steps were carried out at room temperature in 100 mL of the respective buffer.

Surface-based Fluorescence Assay. 250 μL of 0.1 μM Cy3-labeled aptamer in Hybridization Buffer was heated to 72˚C for 5 min, 41˚C for 5 min, and then incubated at room temperature (25˚C) for 45 min. ACE microarrays were hybridized for 18 h at room temperature using custom hybridization chambers in a dark and humidity-saturated container. After hybridization, arrays were dissembled in 4xSSC, followed by washes with 2xSSC for 3 min and 1xSSC for 1 min, all at room temperature. Finally, microarrays were dried using filtered nitrogen. Slides were scanned (Calibration Scan), and stored dry under nitrogen in the dark at room temperature until carrying out an assay.

Hybridized slides were split into identical and addressable sub-arrays using a 16-well gasket (ProPlate 16-well slide module, Grace Bio-Labs, Bend, Oregon, USA). To carry out a surface-based fluorescence assay, ATP (or GTP) was serially diluted in Assay Buffer, and 110 μL of ligand (or buffer only) was added to each sub-array (generating test subarrays and "Buffer" subarrays), except for any "Blank" subarrays, which were not incubated with any buffer. After sample addition, wells were sealed and the slide was incubated for 1 h at room temperature. After sample incubation, slides were dissembled in room temperature 2xSSC and washed in room temperature 1xSSC for 1 min, dried using filtered nitrogen, and scanned (Assay Scan).

In support of the high accuracy, specificity and quantitative nature of measurements made using the surface-based fluorescence assay developed here, it is important to note that the performance of the current assay is superior to a previously published DA for ATP, in which DAs constructed on a glass surface were unable to provide quantitative readouts for ATP[4]. To our knowledge, the study by Carrasquilla *et al.*[4] is the only previous example of a glass- and fluorescence-based ATP DA available in the literature.

Slide Imaging and Microarray Signal Quantification. Slides were scanned in red and green channels using a microarray scanner (G2505C, Agilent Technologies, Santa Clara, California, USA) with a single pass at 5 µm resolution. Microarray features were extracted from the 16 bit TIFF files using Array-Pro Analyzer software (Media Cybernetics, Rockville, Maryland, USA). Data extracted from the green channel for each microarray was analyzed and plotted using custom MATLAB (R2012b, MathWorks, Natick, Massachusetts, USA) and GraphPad Prism (Version 6.0e, GraphPad Software, La Jolla, California, USA) scripts. All data from the solution-based FRET assays and surface-based fluorescence assays were fit using sigmoidal, 4-parameter logistic curve fits (GraphPad Prism).

Data analysis made use of two normalization techniques. First, the fluorescence values of each microarray feature in the Assay Scan were normalized to their corresponding intensity in the Calibration Scan. This allows us to account for slide surface inhomogeneity and any variation in the printing process, and the normalized data can be interpreted as representative of the fluorescence loss occurring during the Assay incubation. Second, fluorescence values for each ACE (e.g. all $5'_{\text{-5C:12}}$ ACE features) were further normalized to the mean fluorescence value of that ACE in the "Blank" sub-array, which itself was defined as having a relative fluorescence value of 1.0 after the Assay incubation step. This second normalization allows for relative changes in fluorescence caused by the presence of a target molecule for each ACE-based DA to be independently assessed, and also normalizes the dataset for any handling-specific changes to DA fluorescence over the slide surface. In this manner, it is possible to quantitatively assess the loss of aptamers from the surface that arise from incubation in buffer-only or target-containing conditions. Finally, for some microarrays a Cy3-labeled control oligonucleotide was also printed in each sub-array, to verify that no differences in inter-subarray fluorescence losses occurred over the course of a surface-based fluorescence assay.

Predicted Thermodynamic Properties. Melting temperatures of hybridized ACEs were calculated using the DINAMelt[5] webserver using the "Hybridization of two different strands of DNA or RNA" application, assuming 200 nM concentrations of each DNA oligonucleotide, Assay Buffer conditions (300 mM Na, 5 mM Mg), and including only the hybridizing portion of the ACE sequence (Not including the surface linker). The melting temperatures reported here were calculated as the temperature at which half of the aptamer molecules are no longer duplexed with an ACE. Other thermodynamic properties were calculated using the "Two-state melting (hybridization)" application using the same conditions (400 nM total strand concentration) at 25˚C; representative secondary structures of DA states are depicted in Fig. 2; a correction value of 7.6 kJ $mole^{-1}$ (a value derived for FAM:BHQ1 interactions[6]) was applied to $\Delta G^{25^\circ C}$ for the predicted K_{Hyb} values, to account for the added stability of the BHQ2:Cy3 dye pair on ACE:aptamer hybridization affinity.

Analytical Model Derivations

1) Apparent aptamer affinity.

In equilibrium-based experiments, the observed affinity of a nucleic acid aptamer for its cognate ligand is equal to the apparent affinity of the aptamer (K_d^{Apt}). This apparent affinity is a function of both the ratio of folded:free aptamer states (Switched ratio, K_S = [Folded]/[Free]), as well as the intrinsic affinity of the fully-folded aptamer for the ligand (K_d^{Int}) (e.g. [6]):

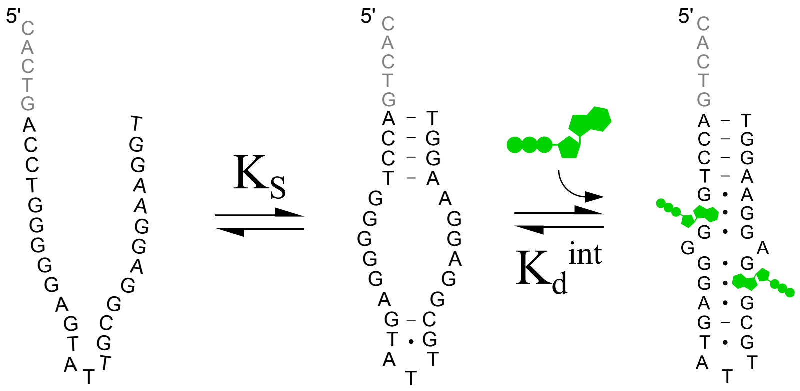

$$K_d^{Apt} = K_d^{Int}(1 + 1/Ks) = 6\ \mu M$$

Accordingly, in this work we have chosen to model DAs on the basis of K_d^{Apt}, and not K_d^{Int}. This is reflected in Fig. 2.

2) Four state model of DA ligand binding.

The four-state, two-pathway thermodynamic cycle for DAs shown in Fig. 2 can also be shown in compact form:

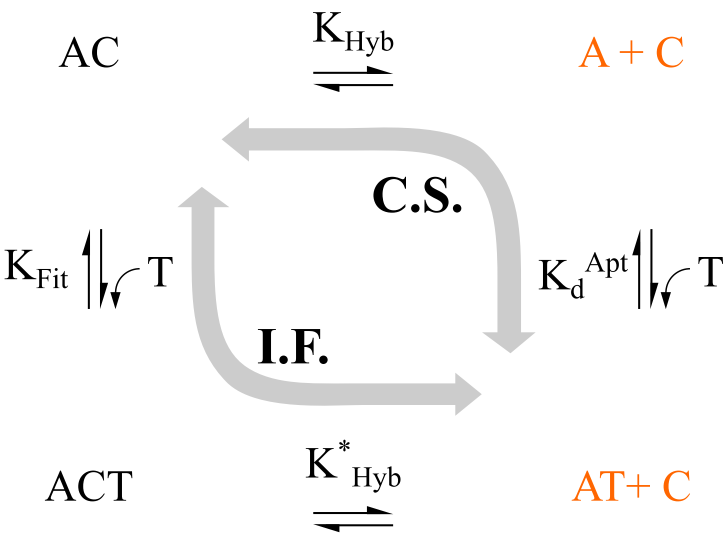

Here, A represents aptamer molecules, C represents ACE molecules, and T represents ligand (ATP) molecules.

The affinity constants for this cycle are defined as:

$$K_{Hyb} = \frac{[A][C]}{[AC]} \; ; \quad K^{*}_{Hyb} = \frac{[AT][C]}{[ACT]}; \quad K_{d}^{Apt} = \frac{[A][T]}{[AT]}; \quad K_{Fit} = \frac{[AC][T]}{[ACT]} \qquad (Eq. 1)$$

At equilibrium, the induced fit and conformational selection pathways in this cycle must have equal equilibrium constants[7], leading to the following equality:

$$\frac{K_{d}^{Apt}}{K_{Hyb}} = \frac{K_{Fit}}{K^{*}_{Hyb}} \qquad (Eq. 2)$$

The apparent affinity (K_d^{app}) of a system is defined as the amount of ligand needed to obtain a half-maximal increase in observed signal from baseline, and can be defined as for a typical affinity constant[7]. For a model of DAs considering only conformational selection, we can define the C.S.-only observed affinity (which we term $K_d^{C.S.}$, Fig. 3c black solid lines) using the effective concentration of reactants (the concentration of A and AC species before T addition, and (ii) the amount of T added) and products (i.e. (iii) the concentration of AT) to arrive at:

$$K_{d}^{C.S.} = \frac{(\,[AC] + [A]\,)\,[T]}{[AT]} \qquad (Eq. 3)$$

Substituting the affinities defined in Eq.1, we have:

$$K_{d}^{C.S.} = K_{d}^{Apt}\left(1 + \frac{[C]}{K_{Hyb}}\right) \qquad (Eq. 4)$$

Likewise, we can consider a model of DAs with only induced fit ligand binding. Here, we use the same logic used to define Eq.3 to define the I.F.-only observed affinity (which we term $K_d^{I.F.}$, Fig. S4b grey solid lines):

$$K_{d}^{I.F.} = \frac{[AC]\,[T]}{[ACT] + [AT]} \qquad (Eq. 5)$$

Substituting the affinity constants defined in Eq.1, we arrive at:

$$K_{d}^{I.F.} = K_{Fit}\left(\frac{1}{1 + \frac{K^{*}_{Hyb}}{[C]}}\right) \qquad (Eq. 6)$$

Finally, we can derive the apparent affinity for a complete model of DAs in which both conformational selection and induced fit are possible. In developing a model of the predicted observed binding affinity of DAs (which we term K_d^{Obs}), we can define K_d^{Obs} as:

$$K_{d}^{Obs} = \frac{(\,[AC] + [A]\,)\,[T]}{[ACT] + [AT]} \qquad (Eq. 7)$$

Substituting the affinity constants defined in Eq.1, we can derive the following equation for K_d^{Obs}:

$$K_d^{Obs} = \frac{K_{Fit}\left(1 + \frac{K_{Hyb}}{[C]}\right)}{1 + \frac{K_{Hyb}^*}{[C]}} \qquad (Eq.8)$$

By substituting for the affinity constants in the equality defined in Eq.2, Eq.8 can be used to predict K_d^{Obs} as a function of either K_{Hyb} or K_{Hyb}^* for known values of K_d^{Apt} and K_{Fit}, as done in Fig. 3c and S4a,b (dotted lines).

Tab. S1 Measured and simulated thermodynamic properties of ACEs tested in this study.

ACE	5'Q$_{-5C:12}$	5'Q$_{-5C:11}$	5'Q$_{-5C:10}$	5'Q$_{-5C:9}$	5'Q$_{-2T:9}$
**$\Delta G_{Exp}^{25^\circ C}$ of hybridized ACE (kJ mole^{-1})**	86.5	74.7	62.4	55.6	57.3
**$\Delta G_{Sim}^{25^\circ C}$ of hybridized ACE (kJ mole^{-1})***	82.9	74.5	66.2	58.6	60.7
**$T_{m,Sim}$ of hybridized ACE (1:1 Q:F ratio) (˚C)**	49.6	42.4	33.6	23.9	29.0
**# bases remaining in ATP-disrupted ACE:aptamer duplex**	7	7	7	7	6
**$\Delta G_{Sim}^{25^\circ C}$ of ACE in weakened duplex (kJ mole^{-1})****	44.0	44.0	44.0	44.0	36.1
**$k^*_{off,Sim}$ for surface-based DAs (s^{-1})*****	0.9	-	-	0.9	22

* For the simulated data, as well as the model presented in Fig. S4a, an additional correction value of 7.6 kJ mole^{-1}, a value derived for FAM:BHQ1 interactions[6], is applied to the simulated $\Delta G^{25^\circ C}$ which is used to calculate K_{Hyb} ($K_{Hyb} = e^{-\Delta G/RT}$) for quencher labeled ACEs, since a Cy3:BHQ2 pair has been found to stabilize duplexes to a similar extent[8,9].

** For the simulated data presented here, as well as the model presented in Fig. S4b, an additional correction value of 7.6 kJ mole^{-1} for the stabilizing Cy3:BHQ2 pair is applied to $\Delta G^{25^\circ C}$ for calculating K^*_{Hyb} for all ATP-disrupted DAs.

*** k^*_{off} values were calculated using $k^*_{off} \equiv K^*_{Hyb} \times k^*_{on}$, and assuming a k^*_{on} of 10^7 M^{-1} s^{-1} given the relatively high salt conditions used here (e.g. [10,11]). K^*_{Hyb} was predicted using DINAMelt with a correction of 3.8 kJ mole^{-1} applied to account for the stabilizing effect of the 5'-Cy3 moiety on the duplex (half the correction applied for a Cy3:quencher pair).

Additional Note: These values represent a lower estimate of duplex stabilities. In particular, in the surface-based assay, the effective salt, aptamer and ACE concentrations are higher, resulting in more stable duplexes on the microarray surface, leading to increased T_m and ΔG values and lower k^*_{off} rates for surface-based DAs.

Tab. S2 Complete sequences of DNA oligonucleotides used in this study.

**Oligo Name**	**Sequence* (IDT Annotation, 5' -> 3')**
Cy3 ATP DNA Aptamer	/5Cy3/ T**CACTG<u>ACCTGGG</u>**<u>GGAGTATTGCGGAGGAAGGT</u>
5'Q$_{-5C:12}$ ACE	**CCCAGGTCAGTG** /3BHQ_2/
5'Q$_{-5C:11}$ ACE	**CCAGGTCAGTG** /3BHQ_2/
5'Q$_{-5C:10}$ ACE	**CAGGTCAGTG** /3BHQ_2/
5'Q$_{-5C:9}$ ACE	**AGGTCAGTG** /3BHQ_2/
5'Q$_{-2T:9}$ ACE	**CCCAGGTCA** /3BHQ_2/
5'$_{-5C:12}$ ACE	/5AmMC12/ TTTTTTTTTTGCTTGTTTTTTTTTT**CCCAGGTCAGTG**
5'$_{-5C:9}$ ACE	/5AmMC12/ TTTTTTTTTTGCTTGTTTTTTTTTT**AGGTCAGTG**
5'$_{-2T:9}$ ACE	/5AmMC12/ TTTTTTTTTTGCTTGTTTTTTTTTT**CCCAGGTCA**

*The consensus aptamer sequence is underlined, and hybridized bases in the aptamer and ACEs are bolded.

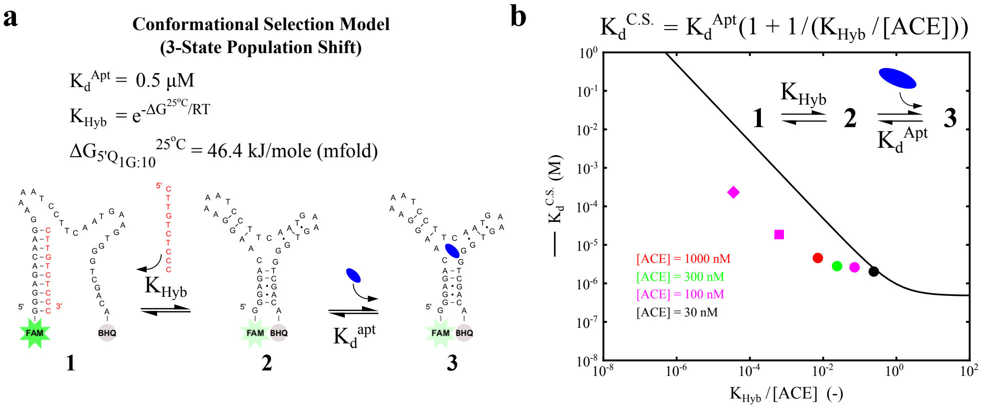

Fig. S1 Graphical representation and modeling of observed binding affinities for cocaine DAs. (a) Overview of cocaine DAs implemented by Porchetta *et al.*, in which a cocaine aptamer (K_d^{Apt} of 0.5 µM) was used to construct DAs using ACEs of 10 to 15 bases in length.[12] Experimentally observed affinities were obtained using a solution-based FRET assay at 25˚C with phosphate buffer (80 mM effective sodium concentration). (b) The experimentally observed binding affinities of the cocaine DAs[12] are qualitatively predicted by a conformational selection-only model ($K_d^{C.S.}$, as derived here), when using DINAMelt to simulate duplex hybridization free energies (46.4, 58.2 and 65.3 kJ mole^{-1} for ACEs of 10 (circles), 12 (squares) and 14 (diamonds) bases). Importantly, although there is some disagreement, the ACEs tested likely form weaker duplexes with the cocaine aptamer (which has a relatively stable secondary structure) than predicted by DINAMelt, and this would bring the model into better agreement with the data.

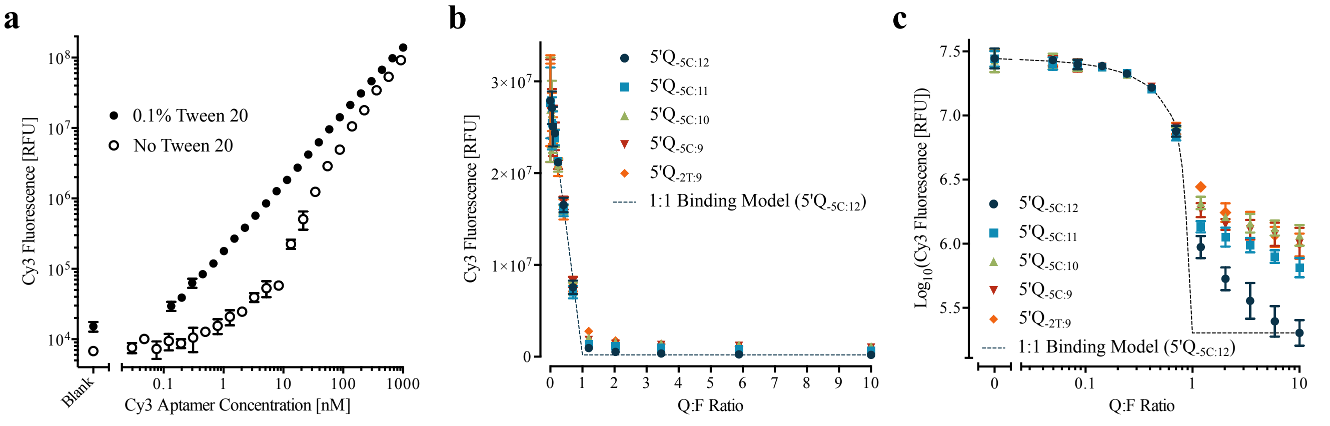

Fig. S2 Additional experimental results of solution-based FRET assay. (a) Linear fluorescence calibration curve for the Cy3 aptamer in Assay Buffer, with and without 0.1 % Tween 20 supplemented. The addition of Tween minimized the absorption of aptamer molecules to plasticware, resulting in improved linearity. (b,c) The degree of Cy3 fluorescence quenching achieved by each ACE was tested using Q:F ratios of up to 10:1, as shown in (b) linear:linear and (c) log:log plots. A 1:1 binding model (dotted line) that assumes an infinite hybridization affinity of the $5'Q_{-5C:12}$ ACE is included as a reference. All ACEs resulted in near-complete quenching above a 1:1 Q:F ratio. The observed quenching efficiencies of both 9mer ACEs were similar, likely due to similarities in the magnitude of FRET- and contact-based quenching for the Cy3-BHQ2 pair[8]. Error bars represent +/- 1 S.D. ($N_{Replicates}$ = 3).

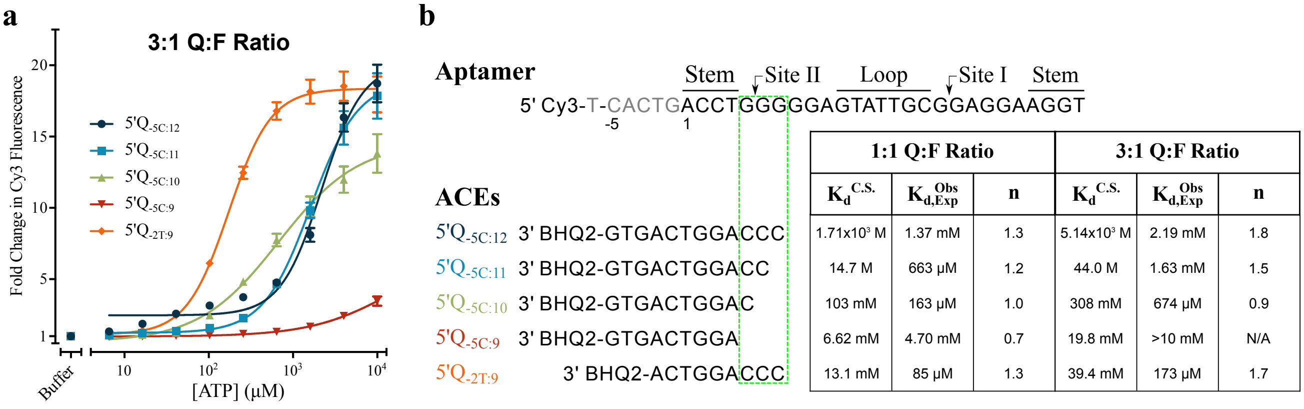

Fig. S3 Experimental results of a solution-based FRET assay using a 3:1 Q:F ratio. (a) Experimental results for 5 ACEs. Lines represent 4 parameter logistic fits used to calculate observed affinities and cooperativities; error bars represent +/- 1 S.D. ($N_{Replicates} = 3$). (b) Schematic of location of ACE hybridization, with table summarizing predicted DA affinities based on conformational selection only ($K_d^{C.S.}$), as well as experimentally observed affinities ($K_{d,Exp}^{Obs}$) and cooperativities (n).

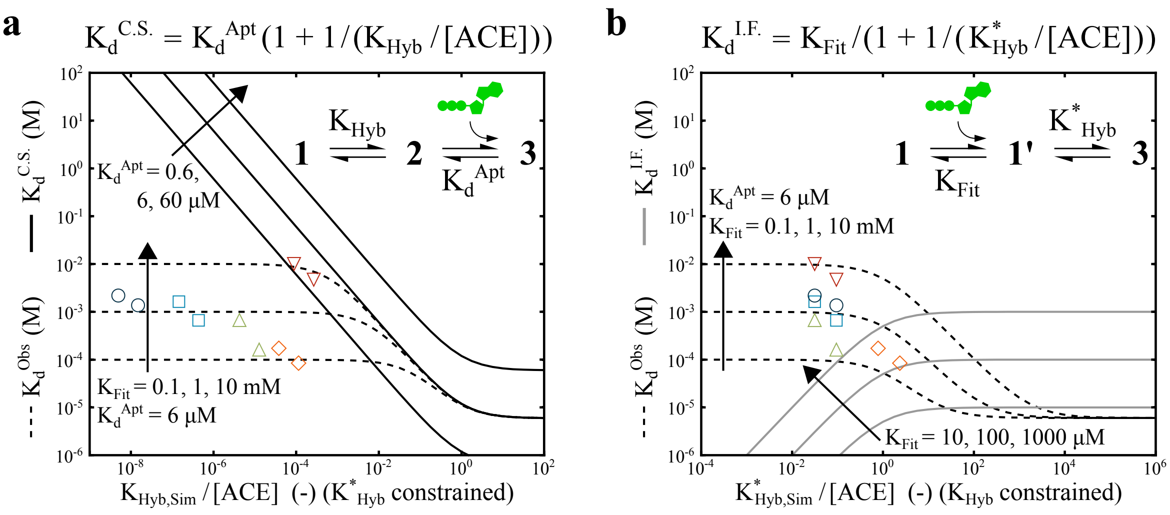

Fig. S4 Additional analytical models of ATP DA observed binding affinities, as a function of simulated K_{Hyb} and K^*_{Hyb} values. (a) Modeling of all $K_{d,Exp}^{Obs}$ values as a function of simulated ACE:aptamer duplex hybridization free energies in the conformational selection pathway (K_{Hyb}/[ACE]), as in Fig. 3c. Solid lines represent a model allowing for only conformational selection binding ($K_d^{C.S.}$ isolines, for varying K_d^{Apt}, black); dashed lines represent a complete model accounting for both conformational selection and induced fit (K_d^{Obs} isolines, for varying K_{Fit}); experimental affinities ($K_{d,Exp}^{Obs}$) from Fig. 3a,b and S3, plotted using simulated K_{Hyb} values. (b) Modeling of all $K_{d,Exp}^{Obs}$ values as a function of the ACE:aptamer duplex hybridization free energy in the induced fit pathway (K^*_{Hyb}/[ACE]). Solid lines represent a model allowing for only induced fit binding ($K_d^{I.F.}$ isolines, for varying K_{Fit}, grey); dashed lines represent a complete model accounting for both conformational selection and induced fit (K_d^{Obs} isolines, for varying K_{Fit}); experimental affinities from Fig. 3a,b and S3, plotted using simulated K^*_{Hyb} values.

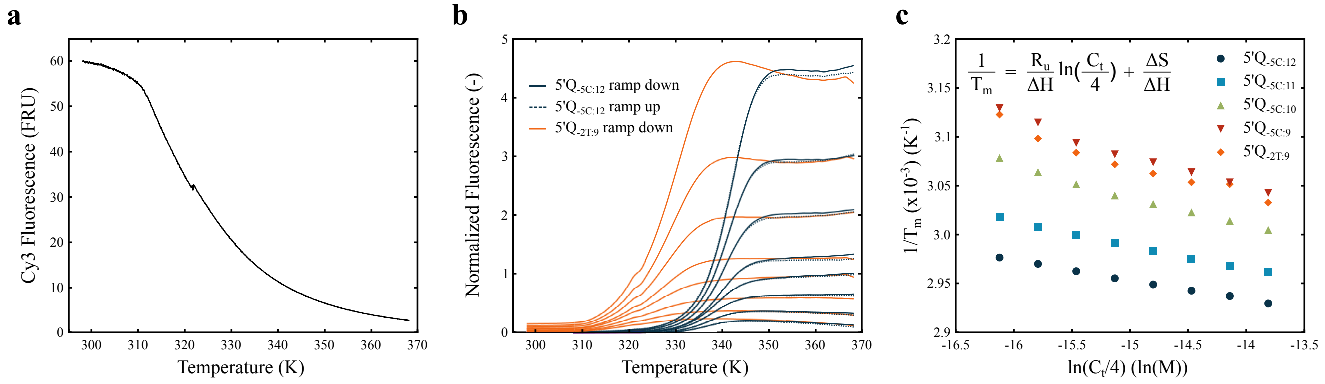

Fig. S5 FRET melting curve experimental results. (a) Temperature dependence of Cy3-labeled aptamer fluorescence. (b) Examples of normalized FRET melting curves for the 5'$Q_{-5C:12}$ (shown for melts ramped both down and up in temperature) and 5'$Q_{-2T:9}$ DAs. (c) van't Hoff plot for all 5 DAs using 8 serial dilutions of 1:1 Q:F ratio. Each data point represents the mean value obtained from two FRET melting curves obtained using temperatures ramped up and temperatures ramped down.

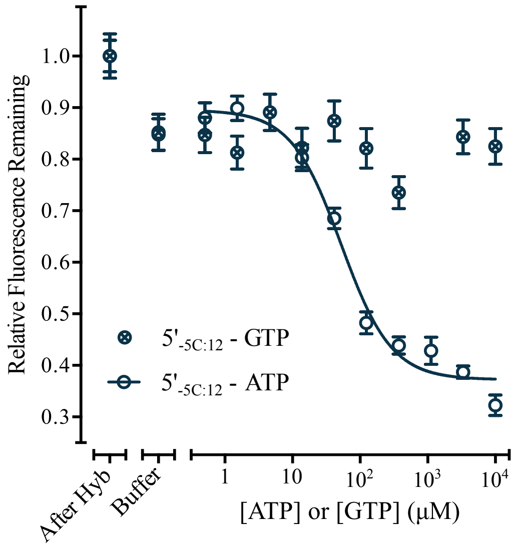

Fig. S6 Experimental (ATP) and negative control (GTP) surface-based fluorescence assays for the 5'$_{-5C:12}$ DA. The 5'$_{-5C:12}$ DA was queried with varying concentrations of either ATP or GTP, as a negative control. Line represents four-parameter logistic fit to the ATP data using GraphPad Prism. Error bars represent +/- 1 S.D. obtained from 10 replicate microarray features per condition for a single surface-based experiment.

References

1 R. Nutiu and Y. Li, *J. Am. Chem. Soc., 2003,* **125**, 4771-4778.

2 R. A. J. Darby *et al.*, *Nucleic Acids Res., 2002,* **30**, e39.

3 P. A. Rachwal and K. R. Fox, *Methods, 2007,* **43**, 291-301.

4 C. Carrasquilla, Y. Li and J. D. Brennan, *Anal. Chem., 2011,* **83**, 957-965.

5 N. R. Markham and M. Zuker, *Nucleic Acids Res., 2005,* **33**, W577-W581.

6 A. Vallée-Bélisle, F. Ricci and K. W. Plaxco, *Proc. Natl. Acad. Sci. U.S.A., 2009,* **106**, 13802-13807.

7 A. Fersht. *Structure and mechanism in protein science: a guide to enzyme catalysis and protein folding*. (Macmillan, 1999).

8 S. A. E. Marras, F. R. Kramer and S. Tyagi, *Nucleic Acids Res., 2002,* **30**, e122.

9 B. G. Moreira, Y. You, M. A. Behlke and R. Owczarzy, *Biochem. Biophys. Res. Commun., 2005,* **327**, 473-484.

10 W. H. Braunlin and V. A. Bloomfield, *Biochemistry, 1991,* **30**, 754-758.

11 S. Howorka, L. Movileanu, O. Braha and H. Bayley, *Proc. Natl. Acad. Sci. U.S.A., 2001,* **98**, 12996-13001.

12 A. Porchetta, A. Vallée-Bélisle, K. W. Plaxco and F. Ricci, *J. Am. Chem. Soc., 2012,* **134**, 20601-20604.
